# Comparative Evaluation of Five Large Language Models for Orthodontic Patient Communication: A Blinded, Factorial Pilot Study

**DOI:** 10.3390/healthcare14213560

**Published:** 2026-10-22

**Authors:** Ferdi Allaf, Mustafa Özcan, Eylül Ögüt Özuğur

**Affiliations:** 1 Department of Orthodontics, Faculty of Dentistry, Istanbul Health and Technology University, 34445 Istanbul, Türkiye; 2 Private Practice (Orthodontist), 34445 Istanbul, Türkiye

**Keywords:** large language models, artificial intelligence, patient education, orthodontics, health communication, ChatGPT, audience adaptation, malocclusion

## Abstract

Background/Objectives: Patients increasingly use large language models (LLMs) to understand orthodontic conditions, yet little is known about how leading models compare on the same patient-communication task or how performance varies with the recipient addressed. Methods: In this blinded pilot, five LLMs—Claude (claude-opus-4-7), GPT (gpt-5.5-2026-04-23), Gemini (gemini-2.5-pro), DeepSeek (deepseek-v4-flash), and Grok (grok-4.3)—each explained three orthodontic malocclusions while adopting three clinician personas and addressing three recipient profiles of differing professional background in a complete balanced 5 × 3 × 3 × 3 design (135 responses in Turkish). Three orthodontists independently scored each response on a seven-axis rubric (1–5). Differences used Kruskal–Wallis, Friedman, and Scheirer–Ray–Hare tests with Holm corrections, epsilon-squared effect sizes, and bootstrap confidence intervals. Results: Inter-rater reliability was good (composite ICC [2, 1] = 0.79). Composite scores differed modestly across models (*p* = 0.044), but no pairwise model difference survived correction; separation was driven by terminology (*p* = 0.005). Among factors, the recipient profile showed the largest effect size (epsilon-squared = 0.058 vs. 0.045 for model); the engineer profile scored lower than a healthcare worker or layperson (both corrected *p* < 0.05), with no statistically significant model × recipient interaction detected. Neither persona nor malocclusion affected quality. Conclusions: Because these were prompted profiles and outcomes were expert-rated, deployment and oversight may benefit from attention to audience fit and terminology rather than model choice alone.

## 1. Introduction

Large language models (LLMs) have quickly become a routine source of health information for the public. People now turn to systems like ChatGPT, Claude, and Gemini to make sense of a diagnosis, weigh treatment options, or get ready for a clinical visit [1]. The shift is especially clear in dentistry, and in orthodontics in particular. Many patients read up on their condition and its side effects online before they ever see a clinician, and model-generated explanations now sit next to conventional patient-education material as they form expectations and make decisions [2,3]. The information patients encounter before a consultation is itself of uneven quality: orthodontist-produced online material on clear aligners has been rated only moderately reliable and often incomplete [4], and orthodontists’ own use of social media for patient communication varies widely [5]. A recent randomized trial in orthodontics went further: a guideline-based chatbot improved patients’ oral-hygiene compliance and treatment knowledge compared with standard leaflets [6]. These tools are no longer a novelty in the clinic.

The growing literature has started to test how well LLMs handle dental patient communication. Orthodontic studies have looked at the reliability and validity of ChatGPT answers regarding treatment and side effects, and more recently at multimodal models for biomechanical reasoning [7]. The general finding is that the output is reasonably accurate but uneven in its completeness and reading level [1,8,9]. Content analyses of model answers on specific topics, such as clear aligners, reach much the same verdict: accuracy is only moderate, and clinically relevant facts are sometimes left out [10,11]. Work in restorative dentistry and other fields shows the same pattern, with answers that read well yet vary in clinical depth and are occasionally misleading [12]. Two limits run through most of this work. The studies usually test a single model, most often a version of ChatGPT, meaning we know little about how the leading models stack up against each other on the same task [1]. And they tend to score generic question-answering and not communication that is deliberately shaped for a particular speaker role or a particular reader.

Newer comparative studies have begun to close the first gap, putting several models side-by-side and using structured instruments and inter-rater statistics to rank them on understandability and readability [3,13,14,15]. A few have also benchmarked model output against expert or standardized answers, and clinician-authored material still tends to come out ahead, especially on completeness and reliability [16]. Together, these studies show that model choice does affect communication quality, but they stay within general dental scenarios. They do not single out the orthodontic malocclusions that drive most real-patient questions, and they do not ask how a model performs when it has to explain the same condition to recipients of differing professional and educational backgrounds.

The second gap, audience- and persona-adaptive communication, is increasingly recognized as central to the safe use of LLMs in healthcare. Conceptually, the promise of LLMs over static pamphlets lies in their ability to tailor an explanation to the reader’s background and literacy [17]. Empirical findings, however, are mixed: audience-specific framing can either improve or degrade the accuracy and usefulness of health information [17]. This makes audience adaptation an open empirical question rather than a solved capability, and it has not been systematically examined in orthodontics, where explanations must bridge substantial gaps between specialist terminology and lay understanding.

Orthodontic malocclusions are a particularly informative test case. Conditions such as open bite, deep bite, crossbite, increased overjet, and Angle Class II and III relationships are common, clinically well defined, and frequently misunderstood by patients. Explaining them well requires accurate terminology, appropriate reassurance, and clear guidance on when to seek care. These are exactly the dimensions on which LLMs are reported to vary. They therefore provide a controlled yet clinically meaningful setting in which to probe which models communicate best and how communication quality shifts across speaker roles and audiences.

As far as we know, no study has looked at these factors together. We therefore ran a factorial evaluation. Five contemporary LLMs each explained orthodontic malocclusions, taking on different clinician personas and addressing recipients of differing professional and technical backgrounds, and three blinded orthodontists scored the output on a structured seven-axis rubric. We set three aims: to compare how the five models perform at patient communication; to measure how quality shifts with the adopted persona and the recipient profile addressed; and to map the axis-level patterns of strengths and weaknesses across models. We expected the models to differ in composite quality, and we expected performance to depend on the persona and audience rather than remaining consistent. This paper reports the pilot phase, which establishes the measurement framework, inter-rater reliability, and early patterns.

## 2. Materials and Methods

### 2.1. Study Design

We conducted a cross-sectional, factorial evaluation comparing the patient-communication performance of five LLMs when explaining orthodontic malocclusions to lay and professional audiences. The design crossed four factors: model, clinician persona adopted by the model, recipient profile, and malocclusion type. Outputs were evaluated by three blinded orthodontist raters using a structured seven-axis rubric. No human participants or patient data were involved; all evaluated text was machine-generated. All prompts and model responses were in Turkish, reflecting the intended clinical setting; the seven-axis rubric and rater instructions were likewise in Turkish.

### 2.2. Language Models Evaluated

Five contemporary LLMs from distinct developers were evaluated via their official APIs using fixed, pinned model versions; the exact identifier returned by each API was logged with each response (Table 1). A sixth candidate (an open-weight Llama-family model) was excluded to avoid confounding from third-party hosting.

### 2.3. Generation Parameters and Prompts

For each design cell, the model was prompted to adopt a specified persona and explain a specified malocclusion to a specified recipient. Prompts were structurally identical and only varied the persona, recipient, and malocclusion slots; each prompt was issued twice. The sampling temperature was 0.7, where supported; two models (Claude Opus 4.7 and GPT-5.5) did not expose a temperature parameter and used provider defaults (a limitation, see Section 4). For Gemini, a maximum output-token budget of 4096 prevented truncated completions.

### 2.4. Factors

Personas (5) included: an orthodontist, a general dentist, a dental assistant/technician, a family physician, and an experienced patient. Recipient profiles (5) included: a healthcare worker, an engineer, a social scientist, an adult with no technical education, and a student. Malocclusions (6) included: open bite, deep bite, crossbite, increased overjet, Angle Class II, and Angle Class III. Of these, a balanced subset was analyzed in this pilot, as detailed in the Pilot Sampling Frame.

### 2.5. Pilot Sampling Frame

The analyzed design is a complete, fully balanced 5 × 3 × 3 × 3 factorial: five models, each evaluated on an identical set of 27 conditions (3 personas × 3 recipients × 3 malocclusions, Supplementary Table S2), for 135 cells in total. The three levels of each factor were fixed a priori during the study design to span a meaningful range while keeping the expert-rating workload feasible for a pilot. Personas spanned a lay-to-specialist gradient (experienced patient, general dentist, orthodontist); recipients represented three distinct communication targets differing in professional and technical backgrounds (healthcare worker, engineer, adult with no technical education); and malocclusions were three common vertical and transverse conditions (open bite, deep bite, crossbite). Two recipient categories considered at the design stage (social scientist, student) were not included; accordingly, our conclusions are restricted to the three evaluated recipient profiles. Each design cell was generated twice, yielding 270 responses in total; the first generation of each cell (135 responses) was carried forward for scoring. This selection rule was fixed a priori and applied by generation order, without reference to response content, quality, or model identity, and the raters were blinded to all of these; therefore, there was no content-based selection. The second-generation responses were retained but were not scored in this pilot; they remain available for the multi-generation analysis planned for the full study. All responses were collected in a single data-collection window without API errors; because commercial models are updated frequently, the pinned versions in Table 1 define the exact systems evaluated.

### 2.6. Outcome Measures

Each response was rated across seven axes on a 1–5 scale: terminology appropriateness (E1), clarity (E2), clinical accuracy (E3), empathy/tone (E4), actionability (E5), persona fidelity (G1), and content validity (G2). The rubric was developed by the research team from the constructs that define effective clinician–patient communication. Each axis was given explicit verbal anchors for the 1, 3, and 5 points to standardize scoring (Supplementary Table S1), and the anchors were refined during the rater calibration round described below. A composite communication score was defined a priori as the mean of the five communication axes E1–E5, which capture communication quality proper. The two remaining axes, persona fidelity (G1) and content validity (G2), were retained as separate control and validity checks rather than as communication-quality dimensions and were therefore analyzed separately, not folded into the composite. The five composite axes were weighted equally, as we had no a priori basis to privilege one communication dimension over another; axis-level results are reported so that the individual contributions remain visible (Table 2).

### 2.7. Rater Procedure and Blinding

Three orthodontists independently scored all responses. Each received an identical, randomized sequence of de-identified responses; the model, persona, recipient, and malocclusion identities were concealed, with only an anonymized code shown. A code-to-factor key was retained solely by the research team. Before independent scoring, raters completed a calibration round on a subset of responses to align interpretation of the rubric anchors.

During the reliability check, one of the raters we first recruited showed almost no rank correlation with the other two on the composite score (Spearman ρ ≈ 0). This pointed to a systematically different reading of the rubric. We excluded that rater’s scores and recruited a replacement. Before scoring independently, the new rater completed a structured calibration round against the same anchor set used for the initial calibration, whose reference values were the consensus scores agreed by the research team. Agreement with the two retained raters was therefore strong (ρ = 0.88 and 0.92). The final analyses rest on these three concordant raters.

### 2.8. Statistical Analysis

Because the axis and composite scores are ordinal ratings on a bounded 1–5 scale, and because the composite departed from normality (Shapiro–Wilk W = 0.97, *p* = 0.003), as did several factor-level distributions, we used nonparametric methods throughout. Inter-rater reliability was assessed for the composite using two-way random-effects intraclass correlation (single-measure ICC [2, 1] and average-measure ICC [2, k]) as interval-approximation indices, since the composite is the mean of five axes and is quasi-continuous. Inter-rater agreement for the discrete 1–5 axis ratings was quantified per axis with an ordinal Krippendorff’s alpha, a chance-corrected multi-rater coefficient that respects the ordering of the scale points [18]; 95% confidence intervals were obtained by bootstrap resampling of texts (5000 resamples). For completeness, we also report per-axis Fleiss kappa as a nominal (ordering-agnostic, and therefore conservative) agreement index. The Kendall W value reported with the omnibus model comparison (Section 3.3) is the Friedman-based concordance statistic for ranking the five models and is not an inter-rater agreement estimate. Model differences in the composite were tested with the Friedman test (blocking unit: the 27 balanced design conditions evaluated across all five models) and, as a complementary marginal test, with Kruskal–Wallis testing. Because the design is fully crossed, pairwise model comparisons used the Wilcoxon signed-rank test, which preserves this pairing, with Holm corrections and rank-biserial r as the effect size. For the between-recipient comparisons, which follow the Kruskal–Wallis omnibus test, pairwise contrasts used Dunn’s test with Holm corrections. The unit of analysis throughout the factorial comparisons was the design cell: each of the 135 cells (one model × persona × recipient × malocclusion combination) contributed a single composite value, namely the mean of the three retained raters’ composite scores for that cell. Because each cell was generated and rated independently and no cell was repeated within a factor level, the 135 cell-level means were treated as independent observations for the between-factor (Kruskal–Wallis) tests. Main effects of the persona, recipient, and malocclusion were evaluated with Kruskal–Wallis tests on these 135 cell-level means, with epsilon-squared as a common effect-size metric (reported in full in Supplementary Table S4) to allow for a direct comparison of factor magnitudes. The matched structure of the design—the same 27 conditions recurring across all five models—was carried by the within-condition pairing in the Friedman and Wilcoxon signed-rank tests used for the model comparison. To probe the model × recipient interaction we additionally applied the Scheirer–Ray–Hare test, a nonparametric analog of two-way ANOVA; because this test assumes independent cell-level observations and does not model the within-condition dependence of the matched design, we treat its interaction result as a screening analysis and interpret it conservatively (see Section 4.7). Consistent with the nonparametric approach, the central tendency is summarized as a median with the first and third quartiles (Q1–Q3); means with bootstrap 95% confidence intervals (5000 resamples) are additionally reported for the principal estimates to convey precision. Axis-level model differences were tested separately. As an objective, rater-independent readability measure, the Turkish Atesman index and words-per-sentence were computed per response. Analyses used Python 3.13 (pandas, NumPy, SciPy, scikit-posthocs).

## 3. Results

### 3.1. Inter-Rater Reliability

The exclusion decision was made at the reliability-assessment stage before any model-level comparison was examined. In transparency, we did not fix a numerical cut-off in advance; the operating rule, agreed before independent scoring, was qualitative: a rater whose composite scores showed essentially no rank association with the other raters (Spearman rho = near zero) would be excluded and replaced. The excluded rater showed near-zero composite rank correlation with each of the other three raters (Spearman rho = 0.01, −0.05, and −0.01; all *p* > 0.58), whereas the three retained raters agreed substantially with one another (pairwise Spearman rho = 0.72, 0.88, and 0.92; all *p* < 0.001). We report these exact post hoc values so that readers can judge the decision directly rather than rely on an implied numerical threshold. One initially recruited rater met this criterion and was replaced by a calibrated rater. The reference for calibration consisted of consensus scores that the three raters agreed on for a small anchor set (approximately 20 responses) during a joint calibration round held before independent scoring. After this discussion, each rater scored the full set independently. After exclusion, replacement, and calibration, agreement across the three retained raters was good for the composite score (ICC [2, 1] = 0.79; ICC [2, k] = 0.92); the reported ICC therefore refers to agreement between the three concordant raters. In a sensitivity analysis retaining the original excluded rater, composite ICC [2, 1] fell from 0.79 to 0.29. We report this sensitivity analysis (Supplementary Table S3) descriptively rather than as justification for the exclusion: the principal findings were unchanged when the excluded rater was retained, with the model ranking preserved (Claude highest) and the recipient effect remaining significant with the engineer profile lowest (Kruskal–Wallis H(2) = 10.08, *p* = 0.007). Axis-level agreement, assessed using ordinal Krippendorff’s alpha across the three retained raters, was highest for terminology appropriateness (E1; alpha = 0.86, 95% CI [0.79, 0.92]) and empathy/tone (E4; 0.78 [0.68, 0.86]); intermediate for clarity (E2; 0.68 [0.56, 0.78]) and actionability (E5; 0.65 [0.53, 0.76]); and lowest for clinical accuracy (E3; 0.53 [0.36, 0.66]), persona fidelity (G1; 0.54 [0.40, 0.67]), and content validity (G2; 0.55 [0.36, 0.70]). The ordering-agnostic Fleiss kappa was correspondingly more conservative on every axis (e.g., E1 kappa = 0.68), as expected for a nominal index applied to ordered categories. All axis scores were averaged across the three retained raters for subsequent analyses.

### 3.2. Overall Performance Across Axes

Composite scores clustered near the scale midpoint. Models performed best on empathy/tone (E4) and actionability (E5) and weakest on terminology appropriateness (E1), indicating warm, action-oriented explanations that were less well-calibrated in technical vocabulary. This pattern was consistent across all five models.

### 3.3. Differences Between Models

Each model was represented by 27 responses. Composite scores differed across models, though the gap was modest (Kruskal–Wallis H(4) = 9.80, *p* = 0.044, epsilon-squared = 0.045; Friedman chi-square(4) = 10.89, *p* = 0.028, Kendall W = 0.10; this W is the Friedman concordance statistic for the model ranking and does not describe inter-rater agreement, which is reported separately in Section 3.3). Claude had the highest composite scores (median 3.40, Q1–Q3 3.00–3.77) and Gemini the lowest (median 3.00, Q1–Q3 2.60–3.40); Grok, DeepSeek, and GPT fell between them (Table 3). In Wilcoxon signed-rank post hoc tests, Claude scored higher than GPT (uncorrected *p* = 0.012, r = 0.34) and Gemini (uncorrected *p* = 0.009, r = 0.34). After Holm corrections, however, none of the pairwise gaps held up (Claude vs. Gemini, Holm-corrected *p* = 0.089; Claude vs. GPT, *p* = 0.106; all other pairs *p* = 1.00), which is what one would expect from a small per-model sample in a pilot.

### 3.4. Axis-Level Differences: Terminology Drives Model Separation

Examined axis-by-axis, the models differed significantly in only one dimension: terminology appropriateness (E1; Kruskal–Wallis H(4) = 14.99, *p* = 0.005, epsilon-squared = 0.085, the largest axis-level effect observed). This was driven by Gemini, which scored markedly lower on terminology (mean 1.81) than the other models (2.36 to 2.96). The remaining axes did not reach significance, although clinical accuracy (E3; *p* = 0.050) and content validity (G2; *p* = 0.051) approached the threshold. The axis-level profile is shown in Figure 1. The modest overall separation between models was therefore attributable chiefly to terminology calibration rather than to broad differences in clinical content or tone.

### 3.5. Effect of Recipient Profile

Among the four factors, the recipient profile showed the largest effect size in this pilot (Kruskal–Wallis H(2) = 9.60, *p* = 0.008, epsilon-squared = 0.058). This exceeded the model effect (epsilon-squared = 0.045) and was far larger than the persona (0.001) or malocclusion (−0.003). We base the description of the recipient profile as the strongest factor on this direct effect-size comparison and not on the contrast in *p* values. Each recipient profile comprised 45 responses. Responses generated for the engineer profile received the lowest composite ratings (median 3.00, Q1–Q3 2.60–3.27; mean 2.85, 95% CI [2.69, 3.00]), significantly lower than those for the healthcare worker (median 3.00, Q1–Q3 3.00–3.40; mean 3.22, 95% CI [3.06, 3.38]; Dunn’s test, Holm-corrected *p* = 0.020) and for the adult with no technical education (median 3.00, Q1–Q3 2.60–3.87; mean 3.28, 95% CI [3.10, 3.46]; Holm-corrected *p* = 0.017), with medium effect sizes by Cohen’s benchmarks (rank-biserial r = 0.32 and 0.33). The persona-by-recipient pattern is displayed in Figure 2. A Scheirer–Ray–Hare analysis detected no statistically significant model × recipient interaction (H = 6.52, df = 8, *p* = 0.59). Given the modest per-cell sample, this absence of a detected interaction should not be read as evidence that the recipient effect is identical across models; it indicates only that no model-specific modulation of the recipient effect reached significance in this pilot. We emphasize that recipients were prompted occupational/educational profiles and not measured health-literacy levels; therefore, the result only supports that responses generated for the specified engineer profile received lower orthodontist ratings in this sample and should not be generalized to technically educated non-medical readers as a population.

### 3.6. Effect of Persona and Malocclusion Type

Neither the adopted persona nor the malocclusion type significantly affected composite quality (persona: H(2) = 2.04, *p* = 0.36, epsilon-squared = 0.001; malocclusion: H(2) = 1.62, *p* = 0.45, epsilon-squared = −0.003). Performance was comparable across speaker roles and conditions. In terms of effect size, the recipient profile (epsilon-squared = 0.058) and model (epsilon-squared = 0.045) were the two influential factors, whereas the persona and malocclusion contributed negligibly.

### 3.7. Objective Readability

We report automated readability descriptively rather than as a test of the expert ratings. Mean Atesman scores (higher = easier) ranged from 54.5 to 69.3, indicating intermediate difficulty; Claude produced the most readable text (69.3; 9.5 words/sentence) and Grok the least (54.5; 12.9 words/sentence). At the individual-response level, however, readability was not associated with the expert clarity (E2) ratings (Spearman rho = 0.06, *p* = 0.48), so readability and expert-judged clarity capture partly distinct aspects of communication. We therefore report the readability scores as an independent, objective description of the texts rather than as confirmation of the clarity ratings.

## 4. Discussion

### 4.1. Principal Findings

This blinded, factorial pilot compared five contemporary large language models on orthodontic patient communication. Overall, the composite quality was similar in magnitude across models, although the omnibus test was significant and we did not perform a formal equivalence analysis; however, three findings stand out. First, differences between models were modest and driven almost entirely by terminology appropriateness rather than by broad differences in clinical content or tone; Claude reached the highest composite mean and Gemini the lowest, whereas after correction, no pairwise model contrast survived, so the ranking is suggestive rather than definitive. Second, among the factors examined, the recipient profile showed the largest effect size, and a formal factorial analysis detected no statistically significant model-by-recipient interaction. Responses written for the prompted engineer profile received lower orthodontist ratings than those for a healthcare worker or layperson. Third, neither the adopted persona nor malocclusion had a measurable effect. We frame the recipient finding cautiously: because we assessed orthodontist ratings of machine-generated text and did not measure patient comprehension, anxiety, or decision quality, the practical implication that terminology adaptation deserves particular attention is a hypothesis for future patient-centered studies rather than a demonstrated patient-safety effect.

### 4.2. Comparison with Prior Work

Most earlier evaluations in orthodontics and nearby dental fields looked at a single model. They found that LLM outputs are generally accurate and readable but uneven in their completeness and reading levels [1,2,8]. Our head-to-head design adds two things. First, when five current models face the same communication task, their overall performance converges rather than splitting into clear tiers. No model was demonstrably better on composite quality once corrections were applied, which echoes the small ranking differences seen in recent multi-model dental comparisons [3, 14,15,,19]. Second, our axis-level view pins down the one place the models truly diverged, terminology, instead of collapsing everything into a single quality score. That distinction matters. Earlier work keeps naming reading level and comprehensibility as the weak spot of LLM patient material [1,12], yet our data suggest the real issue is narrower; that is, matching vocabulary to a particular reader and not overall readability. The results also fit reviews arguing that the main edge of LLMs over static pamphlets is adaptation to the individual reader [17]. What we add here is evidence that this adaptation is still patchy.

### 4.3. Why Terminology Separated the Models

Terminology, the axis that separated the models, may be the one most relevant to patient understanding. Vocabulary pitched at the wrong level can mislead a patient even when the surrounding explanation is warm and well organized; adequate pre-treatment knowledge has been associated with more favorable patient attitudes during fixed orthodontic treatment [20], underscoring why the accuracy and level of the explanation matter clinically. Gemini scored markedly lower here (mean 1.81, against 2.36 to 2.96 for the other models), and this is not a cosmetic gap. It points to a tendency either to use specialist terms without grounding them for the reader, or to oversimplify until precision is lost. Each model scored lowest on terminology yet highest on empathy and actionability. The profile is consistent: current models are reliably reassuring and action-oriented but least dependable exactly where clinical language has to be matched to the listener. Orthodontics makes this especially visible, since its core concepts (overjet, crossbite, the Angle classes) have no everyday equivalents. This is where a clinician reviewing the output adds the most value.

### 4.4. The Recipient Profile Effect

The clearest pattern in this pilot was that expert-rated quality tracked the prompted recipient profile. All five models were rated lower for the engineer profile than for either the healthcare worker or the layperson. The pattern is counterintuitive, since a technically educated reader might seem like an easy audience. Yet it fits the mixed evidence on audience-adaptive generation, where tailoring to a stated reader sometimes helps and sometimes hurts [17]. One plausible explanation is that models are most fluent in two well-practiced registers: a simplified lay explanation, and a peer-to-peer clinical register aimed at someone who already shares the vocabulary. The engineer profile falls between them. Reaching such a reader may well require accurate analogies at a high general-education level, without slipping into clinical shorthand the reader does not have, and without the over-simplification meant for a lay audience. We stress that these were prompted profiles rather than measured literacy levels, so this interpretation is a hypothesis about the specific engineer profile evaluated here and not a demonstrated effect for technically educated non-medical readers as a group.

### 4.5. Implications for Practice and Development

Several practical points follow, all framed cautiously given the pilot design. For clinicians, model choice may matter less than is often assumed for routine explanatory tasks—the five models produced composite scores of broadly similar magnitude; thus, pending a formal equivalence study, costs, availability, and data governance can reasonably inform selection, provided terminology is checked. Notably, the clearest clinical benefit demonstrated so far has come not from an open-ended chatbot but from a guideline-constrained tool evaluated in a randomized trial [6], reinforcing that structured, reviewed deployment matters more than the underlying model. Outputs should also be reviewed with the individual reader in mind. In this sample, the models’ default registers fit least well for the prompted engineer profile specifically; because this profile was an instructed occupational label rather than a measured literacy level, this observation should not be extended to technically educated, non-medical patients as a group. Because tone and actionability were consistent strengths, the residual quality risks concentrated in terminology and content precision, exactly where a brief expert review is most efficient. For developers, the results identify a concrete target: better scaffolding for intermediate-profile readers, and evaluation suites that probe audience adaptation rather than average-case accuracy alone.

### 4.6. Strengths

The study has several strengths. The complete balanced factorial let us examine model, persona, recipient, and condition simultaneously; raters were blinded to all four factors; and a structured, multi-axis rubric with formal reliability statistics let us separate genuine differences from rater noise. Reporting the model × recipient interaction and a rater sensitivity analysis further guards against overinterpretation.

### 4.7. Limitations

Several limitations temper the conclusions. This is a pilot with a modest per-model sample, which limits statistical power; the small sample is the likeliest reason moderate effects did not survive correction, so the model-level findings are hypothesis-generating rather than settled. Each cell was scored from a single (first) generation. The second generation was collected but not independently scored by the raters, so within-cell scoring variability across repetitions could not be quantified in this pilot; therefore, although the selection rule was a priori and content-blind, the LLM output is variable, and scoring both generations is a priority for the full study. The recipient categories were prompted occupational and educational profiles, not validated health-literacy levels, so the recipient finding applies only to the specific profiles evaluated, and two categories considered at design (social scientist, student) and several malocclusions from the wider conceptual space were not evaluated. A temperature asymmetry existed across models, since two systems did not expose a temperature parameter and ran on provider defaults. The model × recipient interaction was screened with the Scheirer–Ray–Hare test, which assumes independent cell-level observations; because the design is matched (the same 27 conditions recur across models), this test does not fully model the within-condition dependence, so its non-significant interaction is a conservative screening result rather than positive evidence that the recipient effect is identical across models, and a repeated-measures or aligned-rank-transform analysis in an adequately powered sample would test the interaction more directly. One initially recruited rater was excluded for near-zero rank correlation and replaced after calibration; we report this openly, and our sensitivity analysis confirms the findings hold with the original rater retained. Finally, all responses were generated and scored in a single language (Turkish), so cross-linguistic generalization is untested, and the results are specific to the pinned model versions. Most importantly, the outcome is orthodontist-rated communication quality, not measured reader comprehension or any patient-centered outcome; whether these rating differences translate into differences in patient understanding, anxiety, or decision quality remains to be tested.

### 4.8. Future Directions

Future work should scale this pilot up to the full factorial design, with a pre-specified power analysis, so the model-level differences can be confirmed or ruled out rather than just observed. Running it across languages would show whether the terminology and recipient effects are general or tied to one clinical vocabulary. That matters for a technology used worldwide. The recipient effect deserves its own study. In particular, future work should test whether the engineer disadvantage can be closed with prompt scaffolding, retrieval augmentation, or explicit reader modeling, and whether it extends to other technically literate non-medical groups. Adding patient-centered outcomes, such as comprehension, anxiety, and decision quality, rather than expert ratings alone would strengthen external validity and tie these measurements to what matters in the clinic, just as recent work directly assessing patient understanding and anxiety has begun to do [21]. And because model capabilities drift with each release, re-running the evaluation on pinned versions from time to time would help separate real gains from version-to-version noise.

## 5. Conclusions

In this blinded, factorial pilot, five leading large language models produced orthodontic patient-communication text of broadly similar composite quality, without a formal test of equivalence, differing mainly in how well they tailored terminology to the reader. Among the factors examined, the prompted recipient profile showed the largest effect size, and a formal factorial analysis detected no statistically significant model-by-recipient interaction. In particular, responses written for the engineer profile received lower orthodontist ratings than those for a healthcare worker or a layperson. Because these were prompted profiles and the outcomes were expert-rated rather than patient-assessed, we frame this as a hypothesis: appropriate deployment and human oversight may benefit from attention to audience fit and terminology precision rather than model choice alone. Larger, more adequately powered multi-generation, multi-lingual, and patient-centered studies are needed to confirm these preliminary patterns and to test whether targeted prompt design can close the gap observed for the engineer profile.

## Figures and Tables

**Figure 1:**
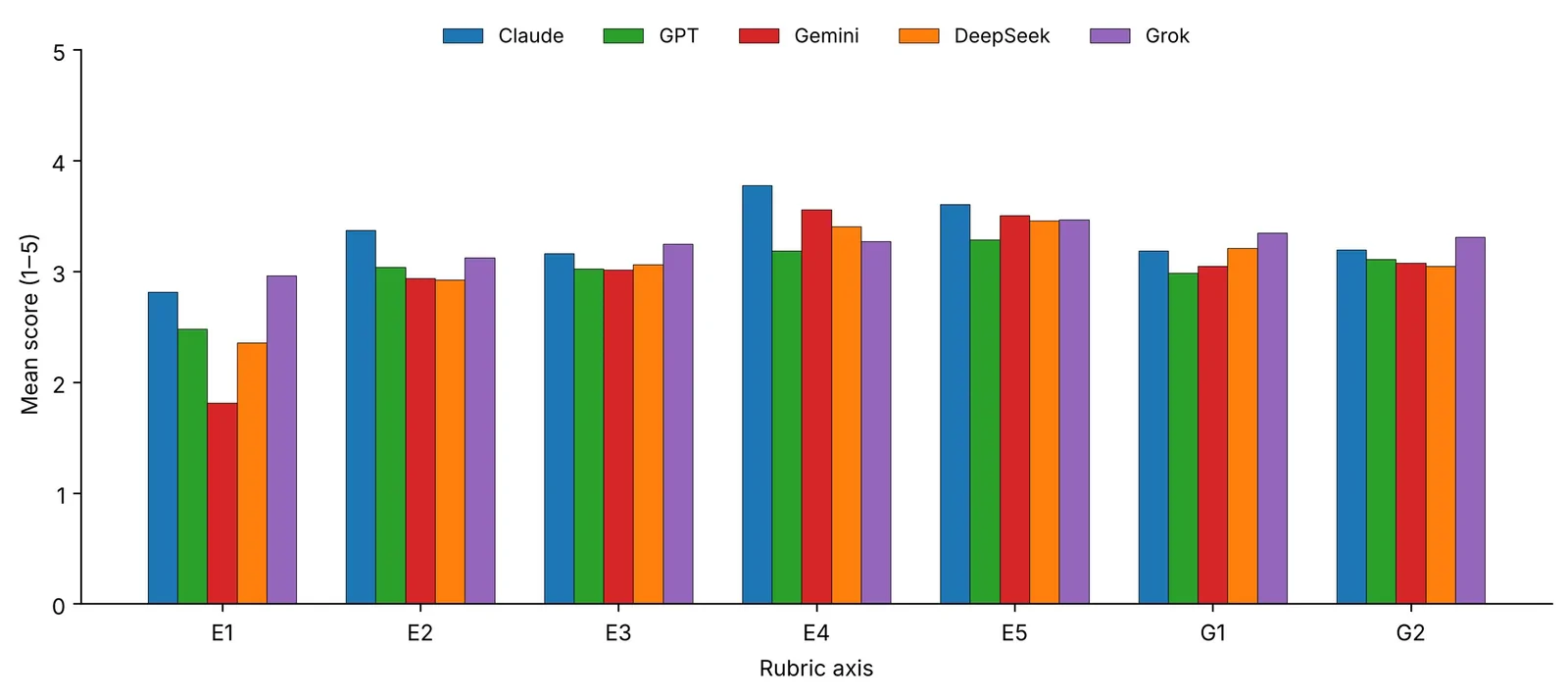
Model × rubric-axis mean scores. Models converge on most axes; Gemini drops sharply on terminology (E1).

**Figure 2:**
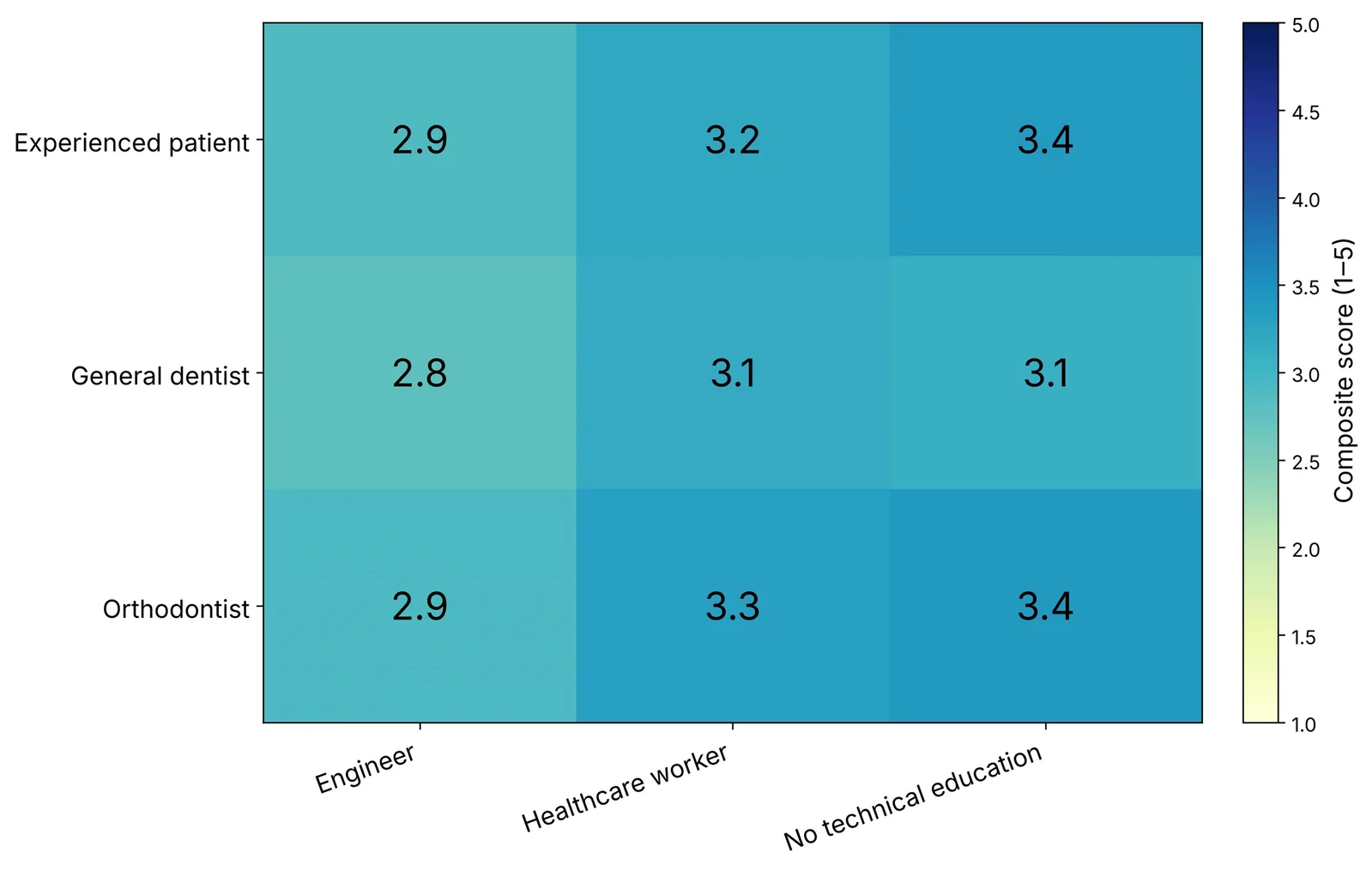
Composite score by persona × recipient profile. The engineer column is consistently the lowest across all personas.

**Table 1:** Evaluated models and pinned API versions.

Model	Developer	API Identifier (Pinned)
Claude	Anthropic	claude-opus-4–7
GPT	OpenAI	gpt-5.5 (gpt-5.5-2026-04-23)
Gemini	Google	gemini-2.5-pro
DeepSeek	DeepSeek	deepseek-v4-flash
Grok	xAI	grok-4.3

**Table 2:** Seven-axis scoring rubric.

Axis	Construct
E1 Terminology	Correctness and audience-appropriateness of terminology
E2 Clarity	Comprehensibility for the target recipient
E3 Clinical accuracy	Factual and clinical correctness
E4 Empathy/tone	Appropriateness and reassurance of tone
E5 Actionability	Clarity of next steps
G1 Persona fidelity	Faithfulness to the stated clinician persona
G2 Content validity	Overall clinical soundness

**Table 3:** Composite communication scores by model.

Model	Median	Q1–Q3	Composite (Mean)	95% CI	SD
Claude	3.40	3.00–3.77	3.35	[3.11, 3.58]	0.63
Grok	3.20	2.87–3.83	3.21	[2.96, 3.46]	0.67
DeepSeek	3.00	2.60–3.40	3.04	[2.79, 3.31]	0.71
GPT	3.00	2.60–3.20	3.00	[2.86, 3.15]	0.39
Gemini	3.00	2.60–3.40	2.97	[2.78, 3.16]	0.53

## Data Availability

The prompt set, de-identified scoring data, and analysis code that support the findings of this study are available from the corresponding author upon reasonable request.

## Supplementary Materials

The following supporting information can be downloaded at: https://www.mdpi.com/article/10.3390/healthcare14213560/s1, Table S1. Seven-axis scoring rubric with anchor definitions; Table S2. Design factors and levels; Table S3. Sensitivity analysis retaining the excluded rater; Table S4. Factor effect sizes (common metric).
