# Supplementary material for: Comparative Evaluation of Five Large Language Models for Orthodontic Patient Communication: A Blinded, Factorial Pilot Study
Source: Healthcare (Basel). 2026 Oct 22;14(21):3560. doi: 10.3390/healthcare14213560 (PMC13763557; doi:10.3390/healthcare14213560)
Supplement: Supplementary file 1 [file healthcare-14-03560-s001.zip › healthcare-4544217-supplementary.pdf]

Supplementary Material

Comparative Evaluation of Five Large Language Models for Orthodontic Patient Communication

Table S1. Seven-axis scoring rubric with anchor definitions

Each response was independently scored 1-5 on each of the seven axes below. Verbal anchors were provided to raters for the 1, 3, and 5 points; 2 and 4 were intermediate. The composite communication score was defined a priori as the mean of E1-E5; G1 (persona fidelity) and G2 (content validity) were retained as separate control/validity checks and analyzed separately. E1-E5 were equally weighted, as there was no a priori basis to privilege one communication dimension over another.

Axis	1 (weak)	3 (moderate)	5 (strong)
E1 Terminology	Terms incorrect or inappropriate	Partly appropriate; some unexplained	Correct, dosed to the recipient
E2 Clarity	Complex, hard to follow	Understandable but occasionally heavy	Fluent, appropriately simple for the target
E3 Clinical accuracy	Incorrect or misleading	Correct but incomplete	Fully correct, complete
E4 Empathy/tone	Cold or anxiety-provoking	Neutral	Reassuring, appropriate to the recipient
E5 Actionability	Unclear	General advice	Clear, followable next steps
G1 Persona fidelity	Not perceptible	Partial	Role distinct and realistic
G2 Content validity	Contains critical errors	Minor errors	Error-free, endorsable

Table S2. Design factors and levels

The analyzed design was a complete, fully balanced 5 x 3 x 3 x 3 factorial (135 cells; each model evaluated on an identical set of 27 conditions). Factor levels were fixed a priori at the design stage.

Factor	Levels (n)	Values
Model	5	Claude, GPT, Gemini, DeepSeek, Grok
Persona	3	Experienced patient, general dentist, orthodontist
Recipient	3	Health care worker, engineer, adult with no technical education
Malocclusion	3	Open bite, deep bite, crossbite

Table S3. Sensitivity analysis retaining the excluded rater

The originally recruited rater showed near-zero rank correlation with the other raters (Spearman rho approximately 0) and was excluded a priori. This table shows that exclusion was warranted and that the principal findings are robust to it.

Analysis	Three retained raters	Four raters (excluded rater retained)
Composite ICC(2,1)	0.79	0.29
Model ranking	Claude highest	Claude highest (unchanged)
Recipient effect	H=9.60, P=.008	H=10.08, P=.007 (engineer lowest, unchanged)

Table S4. Factor effect sizes (common metric)

Epsilon-squared from Kruskal-Wallis tests on the 135 cell-level composite means, allowing direct comparison of factor magnitudes on a common scale.

Factor	H	P	Epsilon-squared
Recipient	9.60	.008	0.058
Model	9.80	.044	0.045
Persona	2.04	.360	0.001
Malocclusion	1.62	.447	-0.003

Model x recipient interaction (Scheirer-Ray-Hare, screening analysis): H = 6.52, df = 8, P = .59. No statistically significant model x recipient interaction was detected; given the pilot sample this does not establish that the recipient effect is identical across models.
